# Urinary Porphyrin Excretion in Neurotypical and Autistic Children

**DOI:** 10.1289/ehp.0901713

**Published:** 2010-06-24

**Authors:** James S. Woods, Sarah E. Armel, Denise I. Fulton, Jason Allen, Kristine Wessels, P. Lynne Simmonds, Doreen Granpeesheh, Elizabeth Mumper, J. Jeffrey Bradstreet, Diana Echeverria, Nicholas J. Heyer, James P.K. Rooney

**Affiliations:** 1 Department of Environmental and Occupational Health Sciences, University of Washington, Seattle, Washington, USA; 2 Autism Research Institute, Lacey, Washington, USA; 3 Autism Society of Washington, Spokane, Washington, USA; 4 Center for Autism and Related Disorders, Tarzana, California, USA; 5 Autism Research Institute and The Rimland Center for Integrative Medicine, Lynchburg, Virginia, USA; 6 International Child Development Resource Center, Melbourne, Florida, USA; 7 Battelle Centers for Public Health Research and Evaluation, Seattle, Washington, USA; 8 Department of Pharmaceutical and Medicinal Chemistry, Royal College of Surgeons in Ireland, Dublin, Ireland

**Keywords:** autism, children, mercury, porphyrins

## Abstract

**Background:**

Increased urinary concentrations of pentacarboxyl-, precopro- and copro-porphyrins have been associated with prolonged mercury (Hg) exposure in adults, and comparable increases have been attributed to Hg exposure in children with autism (AU).

**Objectives:**

This study was designed to measure and compare urinary porphyrin concentrations in neurotypical (NT) children and same-age children with autism, and to examine the association between porphyrin levels and past or current Hg exposure in children with autism.

**Methods:**

This exploratory study enrolled 278 children 2–12 years of age. We evaluated three groups: AU, pervasive developmental disorder-not otherwise specified (PDD-NOS), and NT. Mothers/caregivers provided information at enrollment regarding medical, dental, and dietary exposures. Urine samples from all children were acquired for analyses of porphyrin, creatinine, and Hg. Differences between groups for mean porphyrin and Hg levels were evaluated. Logistic regression analysis was conducted to determine whether porphyrin levels were associated with increased risk of autism.

**Results:**

Mean urinary porphyrin concentrations are naturally high in young children and decline by as much as 2.5-fold between 2 and 12 years of age. Elevated copro- (*p* < 0.009), hexacarboxyl- (*p* < 0.01) and pentacarboxyl- (*p* < 0.001) porphyrin concentrations were significantly associated with AU but not with PDD-NOS. No differences were found between NT and AU in urinary Hg levels or in past Hg exposure as determined by fish consumption, number of dental amalgam fillings, or vaccines received.

**Conclusions:**

These findings identify disordered porphyrin metabolism as a salient characteristic of autism. Hg exposures were comparable between diagnostic groups, and a porphyrin pattern consistent with that seen in Hg-exposed adults was not apparent.

Porphyrins are formed as intermediates in the biosynthesis of heme, a process that proceeds in essentially all eukaryotic tissues. In humans and other mammals, porphyrins with 8,7,6,5, and 4 carboxyl groups are commonly formed in excess of that required for heme biosynthesis and are excreted in the urine in a well-established pattern (Bowers et al. 1992; Woods et al. 1993). In previous studies we described specific changes in the urinary porphyrin excretion pattern (porphyrin profile) associated with prolonged exposure to mercury (Hg) in either organic or elemental forms (Pingree et al. 2001a; Woods et al. 1991, 1993). These changes are characterized by dose- and time-related increases in urinary concentrations of pentacarboxyl (5-carboxyl) and copro- (4-carboxyl) porphyrins and also by the appearance of precoproporphyrin, an atypical porphyrin [molecular weight (mw) = 668] that elutes on high-performance liquid chromatography (HPLC) prior to coproporphyrin (mw = 655) (Woods et al. 1991). The potential utility of these porphyrin changes as a biomarker of Hg exposure and body burden in adults with occupational exposure to elemental mercury (Hg^0^) has been described (Bowers et al. 1992; Gonzalez-Ramirez et al. 1995; Woods 1995; Woods et al. 1993)

Autism (AU), or autistic spectrum disorder (ASD), represents a serious neurodevelopmental disorder that afflicts as many as 1 in 110 children in the United States (Rice 2009). Although genetic factors likely play a principal role in the etiology of autism, a number of studies suggest that environmental exposures, occurring especially at critical periods of neurological development, may trigger events etiologic in AU/ASD among some children. In this respect, several reports (Kern et al. 2007; Mutter et al. 2005; Windham et al. 2006) have implicated prenatal and/or postnatal Hg exposure as associated with autism, in terms of frequency of exposure as well as total body burden. Notably, important mechanistic and toxicokinetic distinctions between different forms of Hg (Burbacher et al. 2005) or in child-specific factors (Faustman et al. 2000) that might affect susceptibility to Hg in autism remain to be fully considered in studies of this association. Nonetheless, some of the neuropsychiatric disturbances associated particularly with Hg^0^ exposure, such as cognition and communication deficits, sensory dysfunction, and impaired motor coordination, are notably similar to those observed in autism and ASD (Echeverria et al. 1998; Kolevzon et al. 2007).

In this context, Nataf et al. (2006) reported that a majority of > 100 French children with clinically confirmed autism displayed a urinary porphyrin excretion pattern comparable with that which we have observed in adult subjects with occupational Hg^0^ exposure and, moreover, that elevated porphyrin levels in these autistic children declined after chelation treatment, also comparable with that seen in occupationally exposed adults (Gonzalez-Ramirez et al. 1995; Woods et al. 1993). Similar findings have been reported among autistic children in the United States (Geier and Geier 2007) and Australia (Austin and Shandley 2008). Although Hg levels or exposure histories of the children involved in those studies were not reported, the precise change in the porphyrin excretion pattern that we observed in association with occupational Hg^0^ exposure in adult subjects implies that exposure to Hg may underlie this response among at least a subset children with autism. A principal concern with respect to these findings, however, is that urinary porphyrin levels in autistic children were commonly evaluated in relation to porphyrin concentrations for older control children or adults, which potentially could be misleading in light of finding that urinary porphyrin concentrations vary substantially with age among children and adolescents (Woods et al. 2009b). Moreover, no reference ranges for all typically excreted porphyrins for children < 8 years of age are currently available. Of additional concern is the common attribution in those studies of elevated porphyrins levels observed among autistic children to increased metal body burden when, in fact, direct measures of metal exposure were not reported.

We undertook the present exploratory study to address several issues associated with the use of urinary porphyrin changes as a diagnostic biomarker of Hg exposure among children and, in particular, those with autism. As the first objective, we measured urinary porphyrin concentrations for neurotypical (NT) children between 2 and 12 years of age against which porphyrin levels in same-age autistic children could be compared. Additionally, we sought to determine if differences in urinary porphyrin levels existed between NT and autistic children of the same age and, if so, if they were consistent with recent Hg exposure as assessed by urinary Hg levels and/or past Hg exposure determined from information acquired online at the time of registration.

## Materials and Methods

### The study population

The principal source of subjects for this study was approximately 600 families with autistic children who subscribe to the informational services of the Autism Research Institute (ARI), Lacey, Washington (USA). We recruited a convenience sample of subjects [NT, AU, and pervasive developmental disorder-not otherwise specified (PDD-NOS) children, ages 2–12 years] into the study via a flyer and sent instructions by the study coordinator at the ARI to all subscribing ARI families, informing them about the study and inviting them to participate. The flyer directed interested parents/caregivers to respond by e-mail or telephone regarding their interest in participating. The study coordinator then contacted interested parents to describe the study and obtain consent. Consenting participants were asked to complete an online enrollment form and to provide a urine sample from the child/children in their families. The estimated participation rate for the ARI was 37%.

The online enrollment form contained detailed questions pertaining to the child’s diagnosis, including diagnostic criteria, diagnosing facility, name of diagnosing clinician, month/year of diagnosis, and diagnostic procedure(s) used. Additional questions were asked regarding dietary practices, drug exposures including chelation history, dental amalgam history, and child’s inoculation history. The number of vaccinations was collected as a potential source of Hg exposure; a distinction was made between total vaccinations and vaccinations prior to the year 2002 when thimerosal, a preservative containing an organomercurial moiety, was eliminated from many vaccines. In addition, to estimate maternal exposures to Hg during the 9 months of the index pregnancy, a count of dental amalgam tooth fillings (a potential source of Hg^0^ exposure) and an estimate of fish meals per week (a potential source of methylmercury exposure) for the mother were obtained for this time period.

Urine samples were collected in the home, transferred to the ARI by hand, and assigned a coded identification (ID) number by the ARI. They were then sent to the University of Washington for analysis, identified only by ID number, age, sex, and diagnosis. Data derived from these studies were evaluated in relation to the diagnostic information provided online to establish mean porphyrin levels for NT children and to determine the association of urinary porphyrin concentrations with autism or related neurobehavioral disorders.

To augment the number of subjects for whom porphyrin comparisons could be made, we analyzed porphyrin concentrations in urine samples acquired from an additional 41 subjects recruited through the Center for Autism and Related Disorders (CARD) in Tarzana, California, and 24 subjects, also through the CARD, from the Rimland Center for Integrative Medicine in Lynchburg, Virginia. CARD subjects were restricted to 2- to 12-year-old NT or AU males who had never undergone chelation treatment and were without amalgam dental fillings. CARD subjects were recruited prior to the initiation of the ARI study and hence did not complete the online enrollment questionnaire used by ARI participants. The estimated participation rate for the CARD was 31%.

Methods of subject recruitment as well as timing and manner of urine collection and processing were comparable between the CARD and ARI cohorts, and preliminary analyses of mean urinary porphyrin and Hg levels by subject source indicated no significant differences within age groupings. Therefore, data from both sources were pooled for porphyrin and Hg analyses. Overall, we enrolled 278 children in the study. After 55 children who had been previously chelated were excluded, 197 children were eligible for analysis. Final statistical models were performed using males only and included 59 NT, 59 AU, and 15 PDD-NOS subjects.

### Human subjects considerations

The study protocol was approved by the institutional review boards at the University of Washington and the CARD. Human subjects approval of the ARI was conferred via an individual investigator agreement between the study coordinator at the ARI and the University of Washington. All parents/caretakers gave written consent for themselves and their children prior to enrollment in the study.

### Diagnostic procedures

For children enrolled through the ARI, diagnosis of autism or other neurodevelopmental disorder was performed by established autism diagnostic and treatment centers that included the University of Washington Autism Center, the Seattle Children’s Autism Center [formerly the Autism Spectrum Treatment and Research Center (ASTAR)], and other pediatric neurology clinics throughout the Pacific Northwest. The diagnosis of AU, PDD-NOS, or other disorder at these centers was made using a multidisciplinary approach that combines a clinical evaluation using the *Diagnostic and Statistical Manual of Mental Disorders, 4th Edition, Text Revision* (DSM-IV-TR) (American Psychiatric Association 2000) criteria, along with a psychological evaluation using the Autism Diagnostic Observation Schedule (ADOS) (Lord et al. 2000), and other established diagnostic procedures such as the Autism Diagnostic Interview-Revised (Lord et al. 1994) or the Childhood Autism Rating Scale (Schopler et al. 1993). Verification of AU status by further psychological testing of children enrolled through the ARI was not feasible in this study. However, comparison of dates of AU diagnosis revealed that > 90% of subjects had been diagnosed since 2003, that is, within the immediate 5-year period since inception of this study, supporting continuity in the methods and procedures used and, therefore, homogeneity in the AU diagnosis. These observations further serve to verify the distinction between diagnoses of AU and other neurodevelopmental disorders, particularly PDD-NOS, as the same testing procedures and treatment centers were employed to diagnose PDD-NOS, most also occurring since 2003. No subjects were diagnosed before 2001.

For subjects enrolled through the CARD, all diagnosed children were fully evaluated by a trained psychologist and met the *International Classification of Diseases, 9th Revision* (World Health Organization 1975) and DSM-IV-TR (American Psychiatric Association 2000) criteria for autism. All diagnoses were verified by obtaining copies of the diagnoses and subsequently validated by additional evaluations at the CARD using the ADOS and other diagnostic procedures cited above.

Participants recruited through the ARI were invited to enroll children with a previous diagnosis of autism or other neurodevelopmental disorder as well as their typically developing siblings. Children whose parents responded “No” to the question “Is this child diagnosed with any neurodevelopmental disorder?” were designated NT. However, verification of NT status through further psychological testing of children enrolled through this process was not conducted. NT subjects enrolled through the CARD were children of CARD employees, all of whom were trained observers and aware of their children’s development. In this respect, all children designated as NT met all developmental milestones, had no symptoms of ASD, ADHD (attention deficit hyperactivity disorder), or (other) learning disabilities, and were seen to be performing successfully in school or preschool with normal peer play. Opportunities for misclassification, therefore, were minimal. The possibility exists that siblings may differ from unrelated controls from the same source population in their genetic contribution to specific inherited disorders such as those affecting porphyrin metabolism. We note in this regard that genetic variation in porphyrin metabolism, particularly that affecting urinary porphyrin excretion, is exceedingly rare especially within the U.S. population, affecting, in the case of the most prevalent form, < 1 in 100,000 individuals (0.001%) (Health Grades, Inc. 2010). Although the absence of differences in genetic variance in related and unrelated NT subjects in this study was not verified, it is unlikely that siblings and unrelated controls differed significantly in this respect.

### Procedures for urine collection and measurement of urinary porphyrins, Hg, and creatinine concentrations

Urine samples (~ 50 mL, first or second morning voids when possible) were collected by parents/caregivers in clean glass containers and then transferred to Nalgene Nunc 60-mL, wide-mouth polyethylene bottles with screw-on lids (Item 2106–0002; Fisher Scientific, Seattle, WA). Samples were delivered frozen to the ARI, where they were logged, assigned an ID number, and shipped in batch in frozen ice packs by overnight express service to the University of Washington. A comparable protocol was followed by the CARD. For analyses, a 10-mL aliquot was removed and acidified with 1 N HCl for Hg analysis by continuous-flow, cold-vapor spectrofluorometry (Pingree et al. 2001b). Porphyrins were quantified in the remaining unacidified portion of the urine sample by HPLC-spectrofluorometric analysis, as previously described (Bowers et al. 1992; Woods et al. 1991). Urinary creatinine concentrations were also measured in unacidified urine using a standard colorimetric procedure (Sigma, St. Louis, MO, USA). Urinary porphyrin concentrations were first creatinine adjusted (nanomoles per gram) and then transformed using the natural logarithm because of the wide variation and skewed distribution. Hg values below the detection limit (LOD) (0.02 μg/L) were assigned 
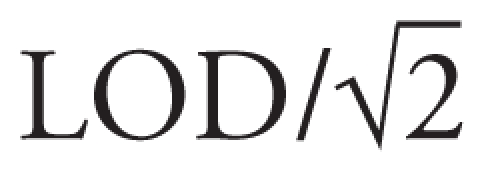
.

### Statistical procedures

Statistical analyses were conducted using PASW Statistics 17.0 (formerly SPSS) (Chicago, IL). Descriptive assessments first eliminated statistical outliers (values ≥ 3 SD in both directions) and then used cross-tabulations and one-way analysis of variance (ANOVA) procedures to compare nonchelated children from the three confirmed diagnostic groups [AU (*n* = 64), PDD-NOS (*n* = 19), and NT (*n* = 114)] with regard to sex, age, potential sources of Hg exposure, and mean (± SD) urinary Hg and porphyrin levels. The small number of five females in the AU group precluded subsequent statistical analyses for each sex. Thus, we examined potential determinants of diagnostic status among only the 133 male children (59 AU, 15 PDD-NOS, and 59 NT).

In males, logistic regression models that controlled for age initially tested potential associations between diagnosis and sources of exposure to Hg from the number of dental amalgam tooth fillings in the child and the mother, the number of vaccines that the child was reported to have received, the number of fish meals per month, and urinary Hg concentrations. The mean (± SD) of each porphyrin was also stratified by diagnosis, age, and sex, where an ANOVA *F*-test was applied separately for each sex to identify statistically significant differences between diagnostic groups.

Logistic regression analyses were also used to evaluate potential associations among males between porphyrins and the risk of having a diagnosis of AU or PDD-NOS, using NT as controls. Statistical measures included regression coefficients, their SDs, and estimates of the strength of association expressed as odds ratios (ORs) and 95% confidence intervals (CIs) for each porphyrin model. A statistically significant association was accepted if *p* < 0.05. Apart from the expected effect of age and age-squared, which were retained in final models, the only covariate that approached statistical significance was a restricted diet (*p* < 0.065). This variable was more reasonably attributed to response to diagnosis rather than to etiology or causal association and therefore was not retained in the analyses. The analyses also evaluated the combination of the three lesser carboxyl porphyrins (hexa-, penta-, and copro-) for potential association with AU or PDD-NOS. Urinary porphyrin concentrations corrected for creatinine (nanomoles per gram), along with the natural logs of these values, were tested in the analyses.

## Results

### The study population

Table 1 describes the demographic distributions for the study population by diagnosis category. Among all children enrolled, 278 had urinary measures of porphyrins and Hg. Among these subjects, 117 were determined to have NT development, 100 met the criteria for AU, and 27 were determined to have PDD-NOS. An additional 34 had other neurodevelopmental diagnoses that included Rett syndrome (*n* = 1), Asperger’s syndrome (*n* = 4), attention deficit hypersensitivity disorder (ADD/ADHD) (*n* = 12), sensory integration disorder (*n* = 5), and language and speech delay (*n* = 3). Fifty-five subjects had undergone chelation therapy, including 3 NT, 36 AU, 8 PDD-NOS, and 8 other. Only the 197 children who had not been chelated and who had diagnoses of NT, AU, or PDD-NOS were included in further analyses, depicted in Table 2. Only five AU and four PDD-NOS cases were girls, consistent with the much lower frequency of autism and related disorders among females. Therefore, although female subjects are included in descriptive analyses of porphyrin levels (Table 3), they were not included in the logistic regression analyses. Thus, logistic regression analyses (Table 4) were conducted among the group of 133 male children that included 59 AU, 15 PDD-NOS, and 59 NT.

As noted in Table 2, age distributions by diagnosis were similar among male subjects. In addition, most covariates were not statistically different between diagnostic groups. In particular, mean urinary past Hg levels, whether unadjusted (micrograms per liter) or adjusted for creatinine (micrograms per gram), were not significantly different between groups. This was also true for the potential sources of Hg exposure, including the mean number of amalgam fillings (both currently in the child or in the mother over the course of pregnancy) and the mean sum of vaccines administered to the child in total, or before 2002.

### Urinary porphyrin concentrations in children

**Figure 1:**
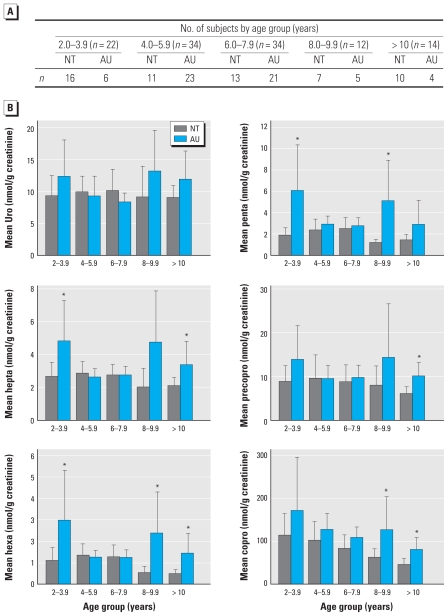
Distributions of urinary porphyrins by age (mean and 95% CI). (*A*) Table describes number of subjects by age group. (*B*) Bar graphs represent mean and 95% CIs of individual creatinine-adjusted urinary porphyrins (nmol/g) by age group for nonchelated NT (*n* = 57) and AU (*n* = 59) boys, age 2–12 years. Graphs depict substantial excess and variable excretion of most porphyrins among AU compared with NT. Porphyrins were evaluated as described under “Materials and Methods.” *NT significantly (*p* ≤ 0.05) different from same-age AU.

**Figure 2:**
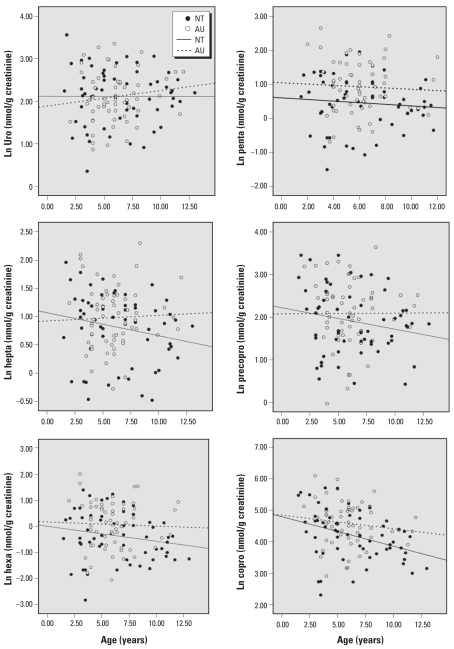
Association between urinary porphyrins and age. Scatterplots and simple linear regression fit lines of natural logs of individual creatinine-adjusted urinary porphyrins by age group for nonchelated NT (*n* = 57) and AU (*n* = 59) boys, age 2–12 years. Graphs clearly depict decreasing porphyrin concentrations with age among NT and disruption of that effect among AU.

The distribution of six urinary porphyrins is presented in Table 3 and Figures 1 and 2. Table 3 presents the mean (± SD) creatinine-adjusted porphyrin concentrations by sex for all nonchelated NT and AU subjects. Values were stratified by 2-year age groups between 2 and 12 years of age. Figures 1 and 2 show the variation in these porphyrin levels by age for males only.

Average concentrations of most porphyrins were elevated in two NT male subjects < 2 years of age compared with older NT males (Table 3). No AU children < 2 years of age were included in the study. Among males in the 2- to 12-year age groups, the mean concentrations of hexacarboxyl- (*p* < 0.01), pentacarboxyl- (*p* < 0.001), and copro- (*p <* 0.009) porphyrins were significantly higher among AU compared with NT groups based on ANOVA *F*-test values, whereas the heptacarboxyl porphyrin was more of borderline significance (*p <* 0.06). Uro- and precoproporphyrins did not differ significantly between AU and NT groups.

We observed substantial variation in creatinine-adjusted urinary porphyrin levels among AU males as well as decreasing concentrations of heptacarboxyl-, hexacarboxyl-, pentacarboxyl-, and coproporphyrins with increasing age among NT males (Figure 1). Scatterplots with simple linear regression fit lines show inverse associations between age and porphyrins among NT males, while also demonstrating that this pattern is disrupted among those with AU (Figure 2).

### Urinary porphyrins and risk of autism

Logistic regression models of age-adjusted associations between porphyrin levels and AU, AU plus PDD-NOS, and PDD-NOS alone (all vs. NT) in males indicated significant associations of hexacarboxyl-, pentacarboxyl-, and coproporphyrins with AU (Table 4). A one-unit increase in the natural log of the creatinine-adjusted value for coproporphyrin is associated with a 2-fold risk for AU (OR = 2.03; 95% CI, 1.15–3.57). Similar associations were observed with pentacarboxyl porphyrin (OR = 2.36; 95% CI, 1.3–4.07) and with hexacarboxyl-, pentacarboxyl-, and coproporphyrins combined (OR = 2.38; 95% CI, 1.42–3.97). In contrast, porphyrin levels did not differ between PDD-NOS and NT males in this study; consequently, combining PDD-NOS and AU subjects weakened associations. Thus, this analysis seems to indicate that AU is a distinct entity from PDD-NOS in terms of being associated with altered porphyrin excretion. Alternatively, too few PDD-NOS subjects were available to this exploratory study to demonstrate an association with PDD-NOS as a less markedly affected portion of the autistic spectrum. Further studies involving greater numbers of subjects with PDD-NOS as well as other recognized disorders of the autistic spectrum are required to determine the extent to which the strength of the association varies with the degree of ASD. Urinary Hg and other Hg-related measures were not significantly associated with AU based on logistic models with and without adjustment for porphyrins (data not shown).

## Discussion

### Urinary porphyrins are naturally elevated in young children

We describe here mean urinary porphyrin concentrations for children in the age range of 2–12 years who participated in the present study. Of particular note is the observation that younger children have inherently higher porphyrin concentrations, particularly of uro-, hepta-, and coproporphyrins, which decline by as much as 2.5 times over the 2- to 12-year age range. Also of interest is the finding that precoproporphyrin, an atypical porphyrin previously identified only in adult humans and animals with prolonged exposure to Hg or Hg compounds, is present in substantial concentrations in urine of younger children. This is a novel and unexpected finding in light of previous observations from studies in animals (Woods et al. 1991) showing that precoproporphyrin is formed as a consequence of Hg inhibition of uroporphyrinogen decarboxylase in the kidney during prolonged exposure, producing excess pentacarboxylporphyrinogen, which then competes with coproporphyrinogen as a substrate for coproporphyrinogen oxidase as the basis of precoproporphyrin formation (Woods et al. 2005). The etiology of this atypical porphyrin in the urine of young children in the presumed absence of prolonged Hg exposure as observed here remains unknown. One possible explanation may be the consequence of accelerated hepatic heme biosynthesis that occurs during the period of perinatal development (Woods 1976). In this respect, formation of precoproporphyrin would be consistent with the observation that the specific activity of hepatic uroporphyrinogen decarboxylase in perinatal rat liver greatly exceeds that of the adult (Woods and Kardish 1983), likely generating comparably greater amounts of pentacarboxylporphyrinogen to compete with coproporphyrinogen as a substrate for coproporphyrinogen oxidase, as proposed in the etiology of precoproporphyrin in the presence of Hg exposure in adults (Woods et al. 2005). Further research is required to confirm this prospect.

### Comparison of urinary porphyrins in NT and AU children

Our findings suggest that mean concentrations of uro- and precoproporphyrins are comparable between NT and AU children of the same age ranges. In contrast, the concentrations of all remaining porphyrins, particularly hexacarboxyl-, pentacarboxyl-, and coproporphyrins, were significantly higher in AU children than NT children, especially in older age groups. Several possibilities might account for these differences. Of initial concern, Hg exposure appears unlikely to play a role in this effect, because no significant differences were observed between NT and AU subjects for indices of past exposure to Hg from dental or medical sources, as reported by parents/caregivers. Additionally, urinary Hg concentrations, measures of recent Hg exposure, were very low among all subjects in this study (Table 2), and no significant differences between diagnostic groups were observed. As noted recently (Woods et al. 2009a), incipient although statistically nonsignificant changes in urinary porphyrin concentrations were seen among children with urinary Hg concentrations derived from prolonged dental amalgam Hg exposure on the order of 3.2 μg/g creatinine. This is nearly 10 times the mean urinary Hg concentration observed among children in this study. Similar findings describing very low blood Hg levels and insignificant differences between NT and AU children have recently been reported (Hertz-Picciotto et al. 2010). These observations do not preclude a possible role of Hg exposure from sources not measured or validated in the present study, especially during the perinatal period, in the etiology of autism or related neurodevelopmental disorders in some children, particularly in relation to genetic variation that may predispose to increased risk of the neurotoxic effects of Hg as Hg^0^ as reported in adults (Echeverria et al. 2005, 2006, 2010; Heyer et al. 2009). Our findings indicate instead that porphyrin metabolism, particularly in preadolescent children, may be too disordered or differently regulated to permit detection of the Hg-mediated changes in urinary porphyrin excretion apparent in adult subjects. Further studies using a substantially larger population, such as the National Children’s Study now in progress (National Children’s Study 2010), are required to resolve this question.

Another factor that may account for the differences in urinary porphyrin levels between AU and NT children is mitochondrial dysfunction, a disorder commonly associated with autism (Correia et al. 2006; Oliveira et al. 2005; Pons et al. 2004). Of particular interest in this respect is the prospect of deficient mitochondrial porphyrin uptake mediated by the recently identified mammalian mitochondrial porphyrin transporter Abcb6 (Krishnamurthy et al. 2006). Abcb6, one of several identified ATP-binding cassette transporters, is located in the outer mitochondrial membrane and has a particularly high affinity for coproporphyrinogen III. Defects in *Abcb6* gene expression or in Abcb6 activity could predispose to impaired mitochondrial porphyrin uptake, leading to cellular accumulation and aberrant porphyrin metabolism and excretion. Similarly, defects in the mitochondrial transmembrane domain that mediates the binding of porphyrins with the Abcb6 transporter could restrict normal porphyrin metabolism, contributing to the disordered porphyrin excretion observed (Krishnamurthy et al. 2006, 2007). Although the association of Abcb6 with autism has yet to be investigated, numerous validated missense mutations of the *Abcb6* gene have been reported (National Center for Biotechnology Information 2010).

Finally, although genetic susceptibility studies were not included as part of the present investigation, previous studies identified a polymorphism in the gene encoding corproporphyrinogen oxidase (CPOX, EC 1.3.3.3) (Li and Woods 2009; Woods et al. 2005) that may predispose to impaired heme biosynthesis and subsequent heme-dependent neurological functions (Chernova et al. 2006; Echeverria et al. 2006). Genotyping studies of 100 DNA samples from autistic children acquired through the University of Washington Autism Center revealed more than double the expected frequency of the homozygous variant of this polymorphism (*CPOX4*) (rs1131857). An intriguing notion rests on the possibility that mitochondrial respiratory chain disorder associated with CPOX4, which itself is linked to the mitochondrial inner membrane (Grandchamp et al. 1978), could account for exaggerated porphyrin excretion as observed here among at least a subgroup of those with autism. Future studies involving a larger cohort of subjects are required to confirm these findings and to define the genetic and/or metabolic factors associated with altered porphyrin excretion in autism.

### Strengths and limitations

A principal limitation of this exploratory study is the relatively small population of NT and AU subjects among whom we sought to define and differentiate excretion levels of metabolites (urinary porphyrins) that can exhibit substantial intraindividual (e.g., diurnal) and interindividual variability, especially in children. Despite this shortcoming, the findings demonstrate significant differences both with age among NT and between NT and AU of the same age, suggesting disordered porphyrin excretion as a metabolic characteristic among at least some AU subjects. Although these findings must be confirmed through larger studies, these preliminary observations provide a context for better understanding and interpreting altered porphyrin excretion among children with AU/ASD.

An additional limitation is potential misclassification of case (AU) or control (NT) status for subjects enrolled through the ARI. We view the possibility of AU misclassification as unlikely, however, because of the multidisciplinary evaluation protocol employed by the neurodevelopmental diagnostic and treatment centers from which subjects were recruited. We note also likely continuity in the diagnostic procedures employed for most subjects enrolled in the study, supporting homogeneity in the AU, PDD-NOS, and NT diagnoses. Nonetheless, our inability to confirm each diagnosis by outside review of subjects’ records is a potential limitation of the present exploratory investigation. Future studies in which subjects are identified through established registries such as the Autism Treatment Network (2010) or that developed by the CHARGE study (Hertz-Picciotto et al. 2006) will minimize prospects of this concern.

Although we made direct measurements of urinary Hg levels as indices of recent Hg exposure, there was the potential for measurement error based on participants’ recall of past Hg exposures from dietary, medical, and dental sources, which could not be fully validated in this study. Such exposures may be of concern in relation to perinatal events that can be accurately assessed only in the context of a prospective study design. Moreover, potential exposures to Hg from other environmental or occupational sources were not assessed and therefore could not be controlled in the present analysis. Thus, although we found few significant differences between AU and NT in reported measures of past Hg exposure, the possibility of exposure misclassification remains a limitation of this study.

Principal strengths of this study were the availability of urine samples for porphyrin and Hg analyses from all study participants and our established capabilities for accurately measuring and interpreting these constituents in the context of this study. Notably, the urinary porphyrin levels reported herein among NT children ≥ 8 years of age are comparable with normative values recently described for children and adolescents of the same ages who were participants in a large clinical trial (Woods et al. 2009b), supporting the generalizability of these findings. The urinary Hg levels measured in this study were also comparable with those reported for a nationally representative sample of children 6–11 years of age acquired as part of the 2003–2004 U.S. National Health and Nutrition Examination Survey [geometric mean = 0.245; 95% CI, 0.213–0.304) (Centers for Disease Control and Prevention 2007). Finally, despite the small number of cases, the results were consistent across age groups with significant differences in porphyrin levels between the diagnostic groups, suggesting that further consideration of this observation may be warranted.

## Conclusions

Mean urinary porphyrin concentrations are inherently high in young children compared with those in adults and decline by as much as 2.5 times between ages 2 and 12 years. Coproporphyrin and heptacarboxyl-, hexacarboxyl-, and pentacarboxylporphyrins were generally elevated among autistic children compared with NT children of the same age. Elevated porphyrin levels among AU children were not associated with measures of past or current Hg exposure, and a porphyrin pattern consistent with that seen in adults with prolonged Hg exposure was not apparent. These findings suggest that disordered porphyrin metabolism may be a salient characteristic of autism and encourage further investigation of genetic, metabolic, and/or environmental factors that may explain this association.

## Note added in proof

Certain drugs that are sometimes administered to autistic children as mood stabilizers or antidepressants, such as valproic acid (depakote, convulex) and risperidone (risperdal), may affect prophyrin metabolism. Participants in this study were selected from among subjects who did not receive such drugs. It is very unlikely, therefore, that such medications contributed to the disordered porphyrin metabolism observed among autistic children.

## Figures and Tables

**Table 1:** Distributions for all subjects.

Total subjects	NT (*n* = 117)	AU (*n* = 100)	PDD (*n* = 27)	Other (*n* = 34)	Total (*n* = 278)
Males	61 (52)	93 (93)	22 (81)	25 (74)	201 (72)
Females	56 (48)	7 (7)	5 (19)	8 (24)	77 (28)
Chelated	3 (3)	36 (36)	8 (30)	8 (24)	55 (20)
Age (years)	6.67 ± 2.96	6.33 ± 2.36	6.88 ± 3.07	7.78 ± 2.60	6.70 ± 2.75

Values are *n* (%) or mean ± SD.

**Table 2:** Demographic and potential risk factors among nonchelated subjects in study.

Nonchelated subjects	Sex	NT (*n* = 114)	AU (*n* = 64)	PDD (*n* = 19)	Total (*n* = 197)
No.	M	59 (52)	59 (92)	15 (79)	133 (67)
	F	55 (48)	5 (8)	4 (21)	64 (33)

Age (years)	M	6.39 ± 3.06	6.01 ± 2.14	6.16 ± 3.03	6.19 ± 2.67
	F	6.93 ± 2.85	4.60 ± 1.82	8.73 ± 4.98	6.86 ± 3.00

Urinary Hg (μg/L)	M	0.29 ± 0.53	0.36 ± 0.62	0.27 ± 0.26	0.32 ± 0.55
	F	0.21 ± 0.45	0.09 ± 0.10	0.30 ± 0.48	0.21 ± 0.43

Urinary Hg (μg/g creatinine)	M	0.28 ± 0.43	0.40 ± 0.66	0.32 ± 0.33	0.34 ± 0.53
	F	0.21 ± 0.48	0.12 ± 0.14	0.29 ± 0.51	0.21 ± 0.46

Creatinine (g/Lr)	M	1.01 ± 0.48	0.95 ± 0.38	0.96 ± 0.41	0.98 ± 0.43
	F	1.03 ± 0.40	0.86 ± 0.30	1.51 ± 0.75	1.04 ± 0.43

Amalgams in child	M	0.18 ± 0.6	0.20 ± 1.1	0.53 ± 1.3	0.25 ± 0.9
	F	0.23 ± 1.2	0.0 ± 0.0	0.0 ± 0.0	0.19 ± 1.1

Amalgams in mother when pregnant	M	2.9 ± 4.2	4.3 ± 5.3	4.0 ± 4.4	3.6 ± 4.6
	F	4.1 ± 4.6	4.6 ± 2.7	3.7 ± 0.6	4.2 ± 4.3

Total vaccines	M	6.5 ± 6.9	10.1 ± 7.8	6.5 ± 6.9	7.9 ± 7.4
	F	8.9 ± 7.1	12.2 ± 3.3	5.8 ± 8.0	8.9 ± 6.9

Vaccines before 2002	M	3.5 ± 5.2	1.8 ± 3.9	1.5 ± 4.1	2.5 ± 4.6
	F	4.1 ± 5.5	2.0 ± 3.5	0.0 ± 0.0	3.6 ± 5.2

Restricted diet (%)	M	24	48	60	41
	F	25	80	100	36

Eat fish (%)	M	59	40	25	55
	F	70	58	53	62

Values are *n* (%), mean ± SD, or %.

**Table 3:** Mean ± SD of porphyrin concentrations (nanomoles per gram creatinine) by NT or AU status, sex, and age (years).

		*n*	Age	Uro	Hepta	Hexa	Penta	Preco	Copro
NT	Male	2	< 2	22.27 ± 18.23	4.49 ± 3.69	0.96 ± 0.47	2.72 ± 1.18	25.18 ± 9.09	250.18 ± 16.16
		16	2–3.9	9.27 ± 6.35	2.66 ± 1.75	1.12 ± 1.18	1.90 ± 1.36	8.84 ± 7.14	114.16 ± 100.83
		11	4–5.9	9.89 ± 4.08	2.86 ± 1.17	1.37 ± 0.87	2.37 ± 1.68	9.56 ± 8.77	102.47 ± 73.40
		13	6–7.9	10.10 ± 6.03	2.74 ± 1.18	1.28 ± 1.00	2.49 ± 1.86	8.75 ± 7.02	83.44 ± 56.32
		7	8–9.9	9.11 ± 6.37	2.01 ± 1.50	0.55 ± 0.39	1.19 ± 0.39	7.94 ± 5.87	62.58 ± 26.26
		10	≥ 10	9.03 ± 2.94	2.09 ± 0.80	0.52 ± 0.27	1.43 ± 0.84	6.05 ± 2.40	46.11 ± 21.99
		59	Total	9.95 ± 6.14	2.60 ± 1.45	1.03 ± 0.92	1.98 ± 1.44	8.93 ± 7.30	92.17 ± 76.61
AU	Male	6	2–3.9	12.31 ± 7.00	4.82 ± 3.02	3.00 ± 2.85	6.07 ± 5.22	13.84 ± 9.46	171.85 ± 152.02
		23	4–5.9	9.24 ± 7.41	2.61 ± 1.25	1.27 ± 0.79	2.91 ± 1.87	9.47 ± 7.13	127.66 ± 89.58
		21	6–7.9	8.31 ± 3.22	2.74 ± 1.22	1.25 ± 0.83	2.75 ± 1.74	9.73 ± 6.35	108.89 ± 55.81
		5	8–9.9	13.14 ± 7.16	4.75 ± 3.50	2.40 ± 2.14	5.10 ± 4.22	14.31 ± 13.70	126.66 ± 86.21
		4	≥ 10	11.87 ± 4.41	3.37 ± 1.43	1.46 ± 0.93	2.89 ± 2.27	10.06 ± 3.09	80.82 ± 28.36
		59	Total	9.73 ± 6.00	3.12 ± 1.88	1.55 ± 1.37	3.36 ± 2.73	10.46 ± 7.58	122.21 ± 84.35
	ANOVA *F*-test	*p*-Value		0.57	0.06	0.01	0.001	0.10	0.009
NT	Female	7	2–3.9	10.65 ± 3.45	2.20 ± 0.58	0.59 ± 0.16	1.94 ± 0.71	8.95 ± 2.55	119.71 ± 44.98
		18	4–5.9	11.79 ± 7.24	2.70 ± 1.77	0.95 ± 1.16	1.67 ± 1.08	8.90 ± 5.00	73.96 ± 41.08
		10	6–7.9	11.25 ± 7.82	2.18 ± 1.25	1.59 ± 2.86	1.53 ± 0.65	5.33 ± 1.98	64.67 ± 44.37
		9	8–9.9	13.93 ± 6.49	3.19 ± 0.91	0.95 ± 0.28	2.05 ± 0.67	9.16 ± 4.13	77.86 ± 41.45
		11	≥ 10	9.48 ± 5.91	2.12 ± 1.10	0.61 ± 0.41	1.34 ± 0.86	4.86 ± 3.49	51.24 ± 31.41
		55	Total	11.43 ± 6.53	2.51 ± 1.34	0.95 ± 1.39	1.67 ± 0.87	7.49 ± 4.21	74.19 ± 43.77
AU	Female	2	2–3.9	20.63 ± 22.04	3.44 ± 3.66	0.61 ± 0.85	1.63 ± 1.61	3.09 ± 0.22	101.60 ± 49.43
		2	4–5.9	14.12 ± 2.76	2.94 ± 1.14	0.89 ± 0.24	1.73 ± 1.09	10.10 ± 4.21	119.87 ± 85.62
		1	6–7.9	17.24	4.04	0.89	1.56	5.69	83.09
		5	Total	17.34 ± 11.57	3.36 ± 1.97	0.78 ± 0.47	1.65 ± 0.97	6.41 ± 4.11	105.20 ± 51.77
	ANOVA *F*-test	*p*-Value		0.07	NS	NS	NS	NS	NS

NS, not significant.

**Table 4:** Logistic regression coefficients and ORs between AU, AU + PDD pooled, and PDD alone (all vs. NT) for urinary excretion of porphyrins among males.

		Porphyrin parameters			
Porphyrin model^a^	Diagnostic group^b^	OR (95% CI)	B	SE	*p*-Value
Uro	AU	1.06 (0.58–1.96)	0.06	0.31	0.84
	AU + PDD	1.04 (0.58–1.86)	0.04	0.30	0.90
	PDD	1.06 (0.42–2.69)	0.06	0.48	0.91

Hepta	AU	1.83 (0.93–3.58)	0.60	0.34	0.08
	AU + PDD	1.57 (0.83–2.97)	0.45	0.32	0.16
	PDD	0.81 (0.30–2.20)	−0.22	0.51	0.67

Hexa	AU	1.65 (1.07–2.55)	0.50	0.22	0.02
	AU + PDD	1.58 (1.05–2.38)	0.46	0.21	0.03
	PDD	1.28 (0.66–2.47)	0.25	0.34	0.47

Penta	AU	2.36 (1.37–4.07)	0.86	0.28	0.00
	AU + PDD	1.86 (1.16–3.00)	0.62	0.24	0.01
	PDD	0.90 (0.42–1.92)	−0.10	0.39	0.79

Preco	AU	1.51 (0.91–2.52)	0.41	0.26	0.11
	AU + PDD	1.18 (0.76–1.82)	0.16	0.22	0.47
	PDD	0.56 (0.27–1.16)	−0.58	0.37	0.12

Copro	AU	2.03 (1.15–3.57)	0.71	0.29	0.01
	AU + PDD	1.68 (1.01–2.80)	0.52	0.26	0.05
	PDD	0.83 (0.38–1.83)	−0.19	0.40	0.65
Hexa/penta/copro	AU	2.38 (1.42–3.97)	0.87	0.26	0.00

a All models include age and age-squared plus the natural log of the creatinine-corrected concentration for individual porphyrins.

b Number of subjects in each diagnostic group: male NT = 59; male autistic = 59; male PDD-NOS = 15.
